# Attitudes toward AI-generated risk prediction in patients with early breast cancer: an international multicenter survey

**DOI:** 10.2340/1651-226X.2025.44030

**Published:** 2025-08-26

**Authors:** Frederik Voigt Carstensen, Sofie A.M. Gernaat, Friederike Banning, Eva Batista, Desiree van den Bongard, Nadia Harbeck, Marle Hattink, Lorenzo Livi, Icro Meattini, Karin Meijer, Jens Petersen, Ivica Ratosa, Helena Verkooijen, Ivan Richter Vogelius, Maja Vestmø Maraldo

**Affiliations:** a Department of Oncology, Copenhagen University Hospital, Rigshospitalet, Copenhagen, Denmark; b Division of Imaging and Oncology, University Medical Center Utrecht, Utrecht University, Utrecht, The Netherlands; c Breast Center, Department of OB&GYN, LMU University Hospital, Munich, Germany; d Breast Unit, Champalimaud Foundation, Lisbon, Portugal; e Department of Radiation Oncology, Amsterdam UMC, Cancer Center Amsterdam, Amsterdam, Netherlands; f Cancer Treatment and Quality of Life/Cancer Biology and Immunology, Amsterdam, Netherlands; g CCC Munich, LMU University Hospital, Munich, Germany; h Department of Experimental and Clinical Biomedical Sciences “M. Serio”, University of Florence, Florence, Italy; i Radiation Oncology Unit & Breast Unit, Azienda Ospedaliero-Universitaria Careggi, Florence, Italy; j Department of Computer Science, University of Copenhagen, Copenhagen, Denmark; k Division of Radiotherapy, Institute of Oncology Ljubljana, Zaloska cesta 2, Ljubljana, Slovenia; l Faculty of Medicine, University of Ljubljana, Vrazov trg 2, Ljubljana, Slovenia

**Keywords:** breast cancer, radiotherapy, artificial intelligence, risk prediction, chronic disease, cardiovascular disease, survey

## Introduction

An estimated 375.000 women in Europe are diagnosed with invasive breast cancer (BC) yearly [1]. Most patients (90%) have early BC and are eligible for curative treatment [2, 3]. Advances in BC care have increased survival, and currently 80% are alive 10 following diagnosis [4]. BC survivors are at increased risk of developing chronic diseases, such as cardiovascular disease (CVD), osteoporosis, interstitial lung disease, and weight gain [5, 6]. The elevated risks are partly due to side effects from chemotherapy, antibody-treatment, radiotherapy (RT), and antihormonal therapies [7, 8].

Most early BC patients receive postoperative RT as part of adjuvant treatment [3]. For RT planning, a computed tomography (CT)-scan is performed, which may harbor information on the patients’ health status but is normally used only for RT planning. The ARTILLERY [9] project aims to develop risk prediction models through trustworthy artificial intelligence (AI) models estimating the risk of common chronic diseases based on RT planning CT-scans of early BC patients. The chronic diseases include CVD, osteoporosis, chronic obstructive pulmonary disease (COPD), and unfavorable body composition. Early identification of early BC patients at high-risk of developing chronic diseases can result in early diagnosis and timely treatment, with a consequent increase in health, quality of life, and survival. In a 2021 study, Gal et al. [10] used a deep learning algorithm to quantify coronary artery calcium (CAC) from planning CT-scans in a cohort of nearly 16.000 BC patients, revealing that elevated CAC scores were significantly associated with increased CVD risk. However, to our knowledge, no prospective studies have used such additional information as the basis for a structured patient intervention. Also, patients’ attitudes toward secondary usage of the RT planning CT-scans have not previously been investigated. The aim of our study is to investigate if early BC patients referred for postoperative RT are interested in such an AI-generated risk prediction.

## Patients/material and methods

The survey was designed as a cross-sectional study including European early BC patients planned for postoperative RT. This study aimed to recruit 40 consecutive participants from nine RT institutions in Denmark (*n* = 3), Netherlands (*n* = 2), Germany (*n* = 1), Slovenia (*n* = 1), Portugal (*n* = 1), and Italy (*n* = 1).

Patients were asked to answer the survey either prior to or directly following their planning CT-scan. Participation was voluntary, and responses had no impact on the patients’ scheduled treatment. Survey data were entered locally into a centralized REDCap (Research Electronic Data Capture) database. The requirement for ethical approval was determined independently by each participating institution in accordance with local regulations and guidelines.

The survey was developed by the ARTILLERY members of the study, in English, and translated locally. Danish BC representatives reviewed the content for clarity, prompting minor revisions.

The survey was administered on paper and consisted of two pages: one with study and ARTILLERY project information, and another with the survey items. The questionnaire included two demographic items (age and smoking status) and four items assessing interest in AI-generated risk predictions for CVD, osteoporosis, COPD, and unfavorable body composition. The response scale was self-constructed, and response options were ‘Yes’, ‘No’, and ‘Don’t know’.

### Data analysis

Descriptive statistics and exploratory multivariate logistic regression were conducted using ‘R for Windows’ version 4.5.0 and ‘R Studio’ version 2024.12.1. The variables included in the multivariate logistic regression were age (continuous), smoking status (binary), country of origin (nominal categorical), and a non-positive attitude toward AI-generated risk predictions (binary).

## Results

**Figure 1:**
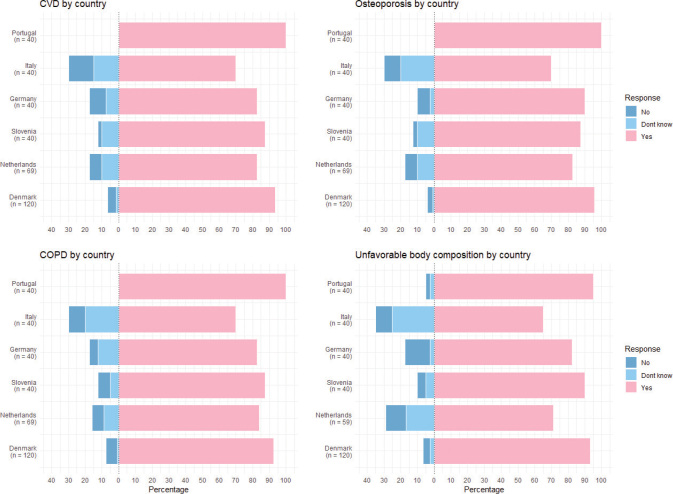
Attitudes toward AI-generated risk prediction of non-communicable diseases shown on country level in percentages.

Data were collected from February 2024 to May 2025. A total of 349 survey responses were collected (Denmark *n* = 120, Netherlands *n* = 69, Germany *n* = 40, Slovenia *n* = 40, Portugal *n* = 40, and Italy *n* = 40). The only variable with missing data was age, which was missing in less than 1% of participants. For all participants, the median age was 59 (range: 28–94) years, and 14.3% (CI95% 10.8–18.4) were active smokers. The majority reported positive attitudes toward a risk prediction, with 87.4% (CI95% 83.4–90.7), 89.1% (CI95% 85.4–92.1), 87.3% (CI95% 83.4–90.7), and 85.1% (CI95% 80.9–88.7) being positive for CVD, osteoporosis, COPD, and unfavorable body composition, respectively. Figure 1 shows the attitudes toward AI-generated risk prediction of chronic diseases by country. Notably, a less positive attitude was observed in Italy, with 70.0% (CI95% 53.5–83.4) answering positively for CVD, 70.0% (CI95% 53.5–83.4) for osteoporosis, 70.0% (CI95% 53.5–83.4) for COPD, and 65.0% (CI95% 48.3–79.4) for unfavorable body composition. Median age for Italian participants was 64 (range: 34–94) years, and 30% (CI95% 16.6–46.6) were active smokers.

Logistic regression was performed to assess associations between age, smoking status, and country, with non-positive attitudes toward AI-generated risk predictions. Older age was significantly associated with non-positive attitudes across all conditions: CVD (OR = 1.05; CI95% 1.02–1.08; *p* < 0.001), osteoporosis (OR = 1.04; CI95% 1.02–1.08; *p* < 0.01), COPD (OR = 1.06; CI95% 1.06–1.10; *p* < 0.001), and unfavorable body composition (OR = 1.03; CI95% 1.00–1.06; *p* < 0.05).

Smoking status was not significantly associated with non-positive attitudes for any of the outcomes.

A significant association was found between Italian nationality and non-positive attitudes toward AI risk predictions for all conditions: CVD (OR = 3.08; CI95% 1.32–6.88; *p* < 0.01), osteoporosis (OR = 3.95; CI95% 1.68–8.96; *p* < 0.01), COPD (OR = 3.03; CI95% 1.28–6.91; *p* < 0.01), and unfavorable body composition (OR = 3.38; CI95% 1.55–7.18; *p* < 0.01).

### Discussion and conclusion

This European multicenter survey demonstrates a strong interest among early BC patients in receiving a predicted risk of developing common chronic diseases using AI. Across the cohort, over 85% of participants expressed a positive attitude, highlighting patients’ acceptance of integrating AI-systems into the standard oncology care pathway.

These findings are particularly relevant, given the high survival rates among early BC patients, which increase the patient awareness of the risks of developing treatment-induced chronic diseases over time. The RT planning CT-scans present an obvious tool for proactive health management, but these imaging datasets remain an underutilized resource.

Notably, a geographical variation was observed, with Italian participants showing comparatively lower interest in AI-based risk predictions. Additionally, Italian respondents had the highest median age and smoking prevalence. While reasons for this discrepancy remain speculative, cultural attitudes toward health technology and varying levels of digital health literacy may contribute. Also, we found that a higher age was associated with a lower interest in AI-generated risk predictions. Understanding factors like age and regional differences can help tailor communication and improve acceptance. These data are valuable for developing patient-centered strategies to implement AI-based risk tools effectively.

Our study was limited by the potential nonresponse bias as the patients who did not want to participate could be more skeptical toward the use of AI or risk predictions. Additionally, the hypothetical nature of the survey items is a limitation, as no information on the performance of the proposed risk prediction models was available to participants.

Overall, these findings support the acceptability of implementing AI-generated risk prediction in clinical oncology settings but highlight the need for patient-centered communication strategies and potential cultural adaptation. Through a prospective trial, ARTILLERY will provide insight into longitudinal outcomes following the implementation of AI risk prediction models, including their impact on early diagnosis of chronic diseases, patient satisfaction, and quality of life.

## Supplementary Material



## Data Availability

The data that support the findings of this study are available from the corresponding author upon reasonable request.
