# Supplementary material for: Attitudes toward AI-generated risk prediction in patients with early breast cancer: an international multicenter survey
Source: Acta Oncol. 2025 Aug 26;64:44030. doi: 10.2340/1651-226X.2025.44030 (PMC12398102; doi:10.2340/1651-226X.2025.44030)
Supplement: Supplementary file 1 [file AO-64-44030-s1.pdf]

Supplementary material has been published as submitted. It has not been copyedited, or typeset by Acta Oncologica

ARTILLERY – Questionnaire

Attitude towards AI-generated risk prediction

AI (artificial intelligence) is a technology where you teach a computer to recognize patterns. In the ARTILLERY project the aim is to investigate if AI can be applied on computed tomography (CT)-scans used for radiotherapy planning to estimate the risk of developing a common disease.

The aim of this questionnaire is to investigate the attitude towards an AI-generated risk prediction among women who will receive radiotherapy for their breast cancer disease.

The AI system is currently under development and is NOT being used in routine clinical care.

The AI systems that will be developed will focus on:
1) Risk of cardiovascular disease
- Calcifications in the coronary arteries and aorta will be detected on CT-scans to estimate the risk of cardiovascular disease. The coronary arteries supply the heart muscle with blood, and calcifications of the coronary arteries are associated with a higher risk of cardiovascular disease, like a heart attack or cardiac arrest.

2) Risk of fractures/osteoporosis
- Bone mineral density (BMD) will be measured on CT-scans to determine the risk of fractures related to osteoporosis.

3) Risk of chronic obstructive pulmonary disease (COPD)
- The lung tissue and the dimensions of the airways will be assessed on CT-scans to look for early signs of COPD and estimate the risk of COPD.

4) Risk of diseases related to unfavorable body composition
- By looking at the distribution of body fat and the amount of fat in the muscle cells, patients with a higher risk of diseases related to unfavorable body composition can be identified.

If the AI-system shows an increased risk of developing one or several of the above-mentioned diseases, the patient will be informed about this risk and counselled accordingly. This may lead to further examinations by healthcare professionals.

Date: Signature:

Age:

Tobacco, active user: YES NO

1) If an AI-system could determine your risk of developing cardiovascular disease, would you be interested in that information?
   YES NO DON'T KNOW

2) If an AI-system could determine your risk of developing a bone fracture due to osteoporosis, would you be interested in that information?
   YES NO DON'T KNOW

3) If an AI-system could determine if your risk of developing COPD, would you be interested in that information?
   YES NO DON'T KNOW

4) If an AI-system could determine your body fat and muscle fat distribution, would you be interested in that information?
   YES NO DON'T KNOW
